# Effects of alkalization therapy on hepatocellular carcinoma: a retrospective study

**DOI:** 10.3389/fonc.2023.1179049

**Published:** 2023-05-29

**Authors:** Masahide Isowa, Reo Hamaguchi, Ryoko Narui, Hiromasa Morikawa, Hiromi Wada

**Affiliations:** Japanese Society on Inflammation and Metabolism in Cancer, Nakagyo-ku, Kyoto, Japan

**Keywords:** hepatocellular carcinoma, cancer metabolism, alkalization therapy, tumor microenvironment, urine pH

## Abstract

**Background:**

In hepatocellular carcinoma (HCC) patients, is difficult to prevent recurrence even when remission is achieved. In addition, even with the advent of drugs that are effective for the treatment of HCC, a satisfactory extension of patient survival has not been achieved. To overcome this situation, we hypothesized that the combination of alkalization therapy with standard treatments will improve the prognosis of HCC. We here report the clinical results of HCC patients treated with alkalization therapy at our clinic.

**Patients and methods:**

Patients with HCC treated at Karasuma Wada Clinic (in Kyoto, Japan), from January 1, 2013, to December 31, 2020 were analyzed. Overall survival (OS) from both the time of diagnosis and the start of alkalization therapy for each patient was compared. The mean urine pH was also calculated as a surrogate marker of tumor microenvironment pH, and OS from the start of alkalization therapy was compared between patients with a mean urine pH of ≥ 7.0 and those with a mean urine pH of < 7.0.

**Results:**

Twenty-three men and six women were included in the analysis, with a mean age at diagnosis of 64.1 years (range: 37–87 years). Seven of the 29 patients had extrahepatic metastases. Patients were divided into two groups according to their mean urine pH after the initiation of alkalization therapy: 12 of the 29 patients had a mean urine pH of ≥ 7.0, and 17 had a mean urine pH of < 7.0. The median OS from diagnosis was 95.6 months (95% confidence interval [CI] = 24.7-not reached), and from the start of alkalization therapy was 42.3 months (95% CI = 8.93-not reached). The median OS from the start of alkalization therapy in patients with a urine pH of ≥ 7.0 was not reached (n = 12, 95% CI = 3.0-not reached), which was significantly longer than that in patients with a pH of < 7.0 (15.4 months, n = 17, 95% CI = 5.8-not reached, *p* < 0.05).

**Conclusions:**

The addition of alkalization therapy to standard therapies may be associated with more favorable outcomes in HCC patients with increased urine pH after alkalization therapy.

## Introduction

The incidence of hepatocellular carcinoma (HCC) in Japan is documented to be 29.6 cases per 100,000 people (2019), with a 5-year survival rate of 35.8% (2009–2011) (1). HCC is known to develop in the background of cirrhosis in patients with underlying irreversible liver diseases (i.e., hepatitis B and C, alcoholic hepatitis, and nonalcoholic fatty liver disease). With the development of antiviral therapies for viral hepatitis, there are now many options for disease control by local therapies, such as surgery, transarterial embolization (TAE), transarterial chemoembolization (TACE), and radiofrequency ablation (RFA). In addition, therapeutic strategies for HCC have changed with the indication of atezolizumab and bevacizumab combination therapy, and the advent of sorafenib, lenvatinib, regorafenib, ramucirumab, and cabozantinib. However, whereas the overall survival (OS) time of patients with HCC has increased (5-year survival, 2001–2007: 19.30% vs. 2008–2013: 22.40%), the most significant survival improvements have been seen in patients with early-stage HCC limited to single lesions or multiple intrahepatic lesions without vascular invasion (2). There has been no significant change in survival rates of patients with late-stage HCC, who have vascular invasion or distant metastases, and HCC remains a clinically difficult disease to manage (2). Furthermore, it is difficult to prevent the recurrence of HCC, and repeated invasive treatments are required in many cases, resulting in a lack of favorable outcomes. Therefore, there is a need for new treatment methods for HCC.

Malignant tumors depend on the glycolytic system for their metabolism, and excrete more protons extracellularly than normal cells. Therefore, the tumor microenvironment (TME) is known to be acidic (3). In addition, acidification of the TME, i.e., a decrease in pH, attracts inflammatory cells and results in chronic inflammation, creating an even more favorable environment for malignant tumors (4). There are reports that reversing this phenomenon, and alkalization of the TME may have anti-tumor effects, such as inhibiting tumor growth and removing the resistance to anti-cancer drugs (5). For example, the acidic microenvironment of the TME may affect processes involving P-glycoprotein and Absorption, Distribution, Metabolism and Excretion, leading to resistance to chemotherapy (6). Current treatment approaches for malignant tumors assumes that therapeutic interventions targeting only the tumor cells themselves will achieve sufficient anti-tumor effects. However, close observation of the behavior of tumor cells has resulted in a crucial paradigm shift, to the idea that not only the tumor cells themselves but also the surrounding environment, i.e., the TME, should be targeted (7). Therefore, we have reframed our treatment strategy for cancer from a molecular biological perspective, and have also reported in two previous papers on our clinical experience that alkalization therapy, mainly based on dietary modification, has strong antitumor effects against various solid tumors and suppresses tumor progression. In a comparison of chemotherapy plus alkalization therapy versus chemotherapy alone for small cell lung cancer at our clinic, the median overall survival (OS) for the combination treatment group was 44.2 months (95% confidence interval [CI] = 22.0-not reached) versus 17.7 months for the chemotherapy alone group (95% CI = 13.5-not reached; *p* < 0.05) (8). In a comparison of chemotherapy plus alkalization therapy versus chemotherapy alone in the treatment of advanced pancreatic cancer, the median OS was 15.4 (combination treatment group) vs. 10.8 months (chemotherapy alone group) (*p* < 0.005) (9). Moreover, we previously found that patients with renal cancer, malignant lymphoma, gastric cancer, and breast cancer who were treated with alkalization therapy had favorable outcomes (10). As it remained unclear whether the combination of alkalization therapy with standard treatments would be as effective for HCC as for the cancers described above, we conducted a retrospective study of the effects of alkalization therapy on patients with HCC. Alkalization therapy in this report is defined as a treatment aimed at alkalizing the entire body using an alkaline diet and alkalizing agents, such as sodium bicarbonate and citric acid (11–13). In addition, the pH of urine is known to change depending on the diet consumed (14, 15). In previous reports, urinary pH has been utilized as a monitoring parameter for whole-body buffering, due to its ease of use, noninvasiveness, and low cost. In the present study, urinary pH was also used as a marker of whole-body pH. An “alkaline diet” is defined as a diet based on vegetables and fruits, with seafood as the main source of protein. Meat and dairy products are listed as foods to avoid (11).

## Patients and methods

### Study design

**Figure 1:**
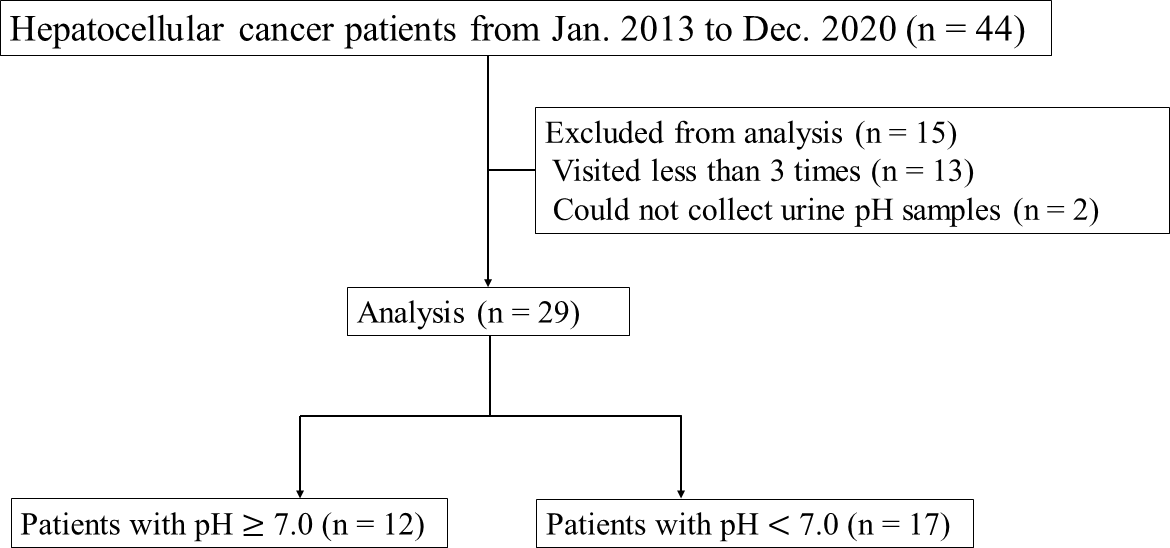
Flowchart of this study. Flowchart showing the number of patients analyzed in this study.

This retrospective study was conducted to investigate the effects of alkalization therapy on HCC patients treated at Karasuma Wada Clinic (in Kyoto, Japan), from January 1, 2013, to December 31, 2020, using the clinic’s medical records. All patients received alkalization therapy (an alkalization diet with oral intake of alkalizing agents) as described below, together with standard treatments for HCC (surgery, TAE, TACE, RFA, chemotherapy, etc.). A flowchart of this study is shown in **Figure 1**. All procedures were performed in accordance with the ethical principles described in the 1995 Declaration of Helsinki. Written informed consent was obtained from each patient. The study was approved by the Institutional Review Board of the Japanese Society of Clinical Oncology, and registered in UMIN Clinical Trials (UMIN000049949).

### Alkalization therapy

Alkalization therapy was defined as a combination of an alkalizing diet and oral bicarbonate (3.0−5.0 g/day) and/or citric acid (3.0−6.0 g/day) therapy. The alkalizing diet was a diet high in vegetables and fruits, and as low in meat and dairy products as possible. All patients who received alkalization therapy were instructed to take at least 400 g of fruit and vegetables a day, and not to take any meat and dairy products, and they recorded their daily meals for at least the first 4 weeks from the start of the alkalizing diet. Their records were reviewed to confirm whether their meals were appropriate or not by a doctor or nurse at every visit, and they were given advice according to their records, but the actual diet was decided by the patients themselves.

### Assessment procedures

The OS from both the time of diagnosis and from the start of alkalization therapy in each patient was calculated. Urine was collected at the patients’ regular visits, which were at least once every two months, and up to twice a month. The mean urine pH of each patient was calculated, and patients were divided into those with a mean urine pH of ≥ 7.0 and those with a mean urine pH of < 7.0. The OS from the start of alkalization therapy was compared between the patients with a mean urine pH of ≥ 7.0 and those with a mean urine pH of < 7.0.

### Statistical analyses

Mean urine pH values for each patient were calculated from all urine pH samples collected, from the patient’s first visit up to December 31, 2022, at Karasuma Wada Clinic. OS from the time of the start of alkalization therapy was calculated using Kaplan–Meier estimates, and compared between patients with a mean urine pH of ≥ 7.0 and those with a mean urine pH of < 7.0. Standard deviations of mean dataset values were calculated. All *p*-values were two-sided, and a *p*-value of less than 0.05 was considered to indicate a statistically significant difference between two groups. All statistical analyses were performed with Easy R (version 1.61; Saitama Medical Center, Jichi Medical University, Saitama, Japan), which is a graphical user interface that is a modified version of R (The R Foundation for Statistical Computing, Vienna, Austria) (16).

## Results

### Patient characteristics

Forty-four patients with HCC visited Karasuma Wada Clinic between January 1, 2013, and December 31, 2020. Of these patients, 13 who visited fewer than three times, and two for whom urine samples could not be collected were excluded. The patients analyzed comprised 23 men and six women, and the mean age at diagnosis was 64.1 (range = 37–87) years. All patients were treated with alkalization therapy. Seven patients out of the 29 had extrahepatic metastases. Background liver diseases and standard therapies that the patients received are presented in **Table 1**.

**Table 1:** Patient characteristics.

	All patients
No. of patients	29
Age (range), years	64.1 (37–87)
Sex	
Men	23
Women	6
Background liver disease	
HCV	13
HBV	4
Alcoholic hepatitis	2
Others	10
Mean urine pH after alkalization therapy	6.71 ± 0.57
Extrahepatic metastases	
Yes	7
No	22
Surgery	
Yes	11
No	18
RFA	
Yes	14
No	15
TACE/TAE	
Yes	17
No	12
Drug therapy	
Yes	20
No	8
Unknown	1

RFA, radiofrequency ablation; TACE, transcatheter arterial chemoembolization; TAE, transcatheter arterial embolization; HCC, hepatocellular carcinoma; HCV, hepatitis C virus; HBV, hepatitis B virus.

All urine pH data up to December 31, 2022 were collected, and the mean urine pH was calculated for each patient after alkalization therapy. Patients were divided into two groups according to their mean urine pH after the start of alkalinization therapy. Twelve patients out of the 29 had a mean urine pH of ≥ 7.0, and 17 out of the 29 had a mean urine pH of < 7.0. Patient characteristics of both groups are presented in **Table 2**.

**Table 2:** Characteristics of patients with a mean urine pH ≥ 7.0 and those with a mean urine pH < 7.0.

	Patients with mean urine pH ≥ 7.0	Patients with mean urine pH < 7.0
No. of patients	12	17
Age (range), years	62.3 (37–87)	65.3 (50–80)
Sex		
Men	7	16
Women	5	1
Background liver diseases		
HCV	5	8
HBV	2	2
Alcoholic hepatitis	0	2
Others	5	5
Mean urine pH after alkalization therapy	7.23 ± 0.20	6.43 ± 0.45
Extrahepatic metastases		
Yes	4	3
No	8	14
Surgery		
Yes	7	4
No	5	13
RFA		
Yes	10	4
No	2	13
TACE/TAE		
Yes	7	10
No	5	7
Drug therapy		
Yes	8	12
No	4	4
Unknown	0	1

RFA, radiofrequency ablation; TACE, transcatheter arterial chemoembolization; TAE, transcatheter arterial embolization; HCC, hepatocellular carcinoma; HCV, hepatitis C virus; HBV, hepatitis B virus.

### OS between patients with different urine pHs

**Figure 2:**
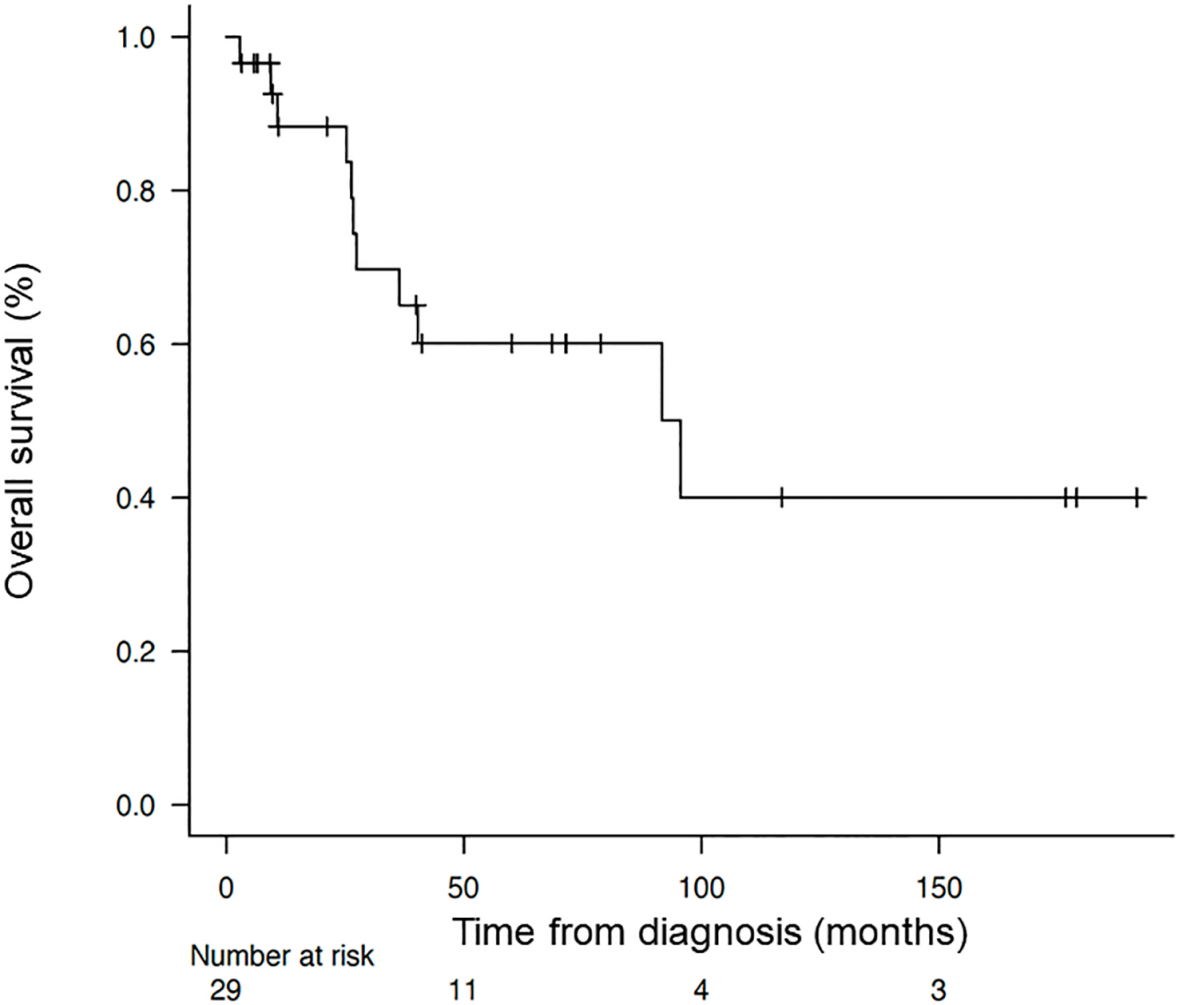
Overall survival of the patients from the time of diagnosis. Kaplan–Meier curve of the overall survival of the patients from the time of diagnosis.

**Figure 3:**
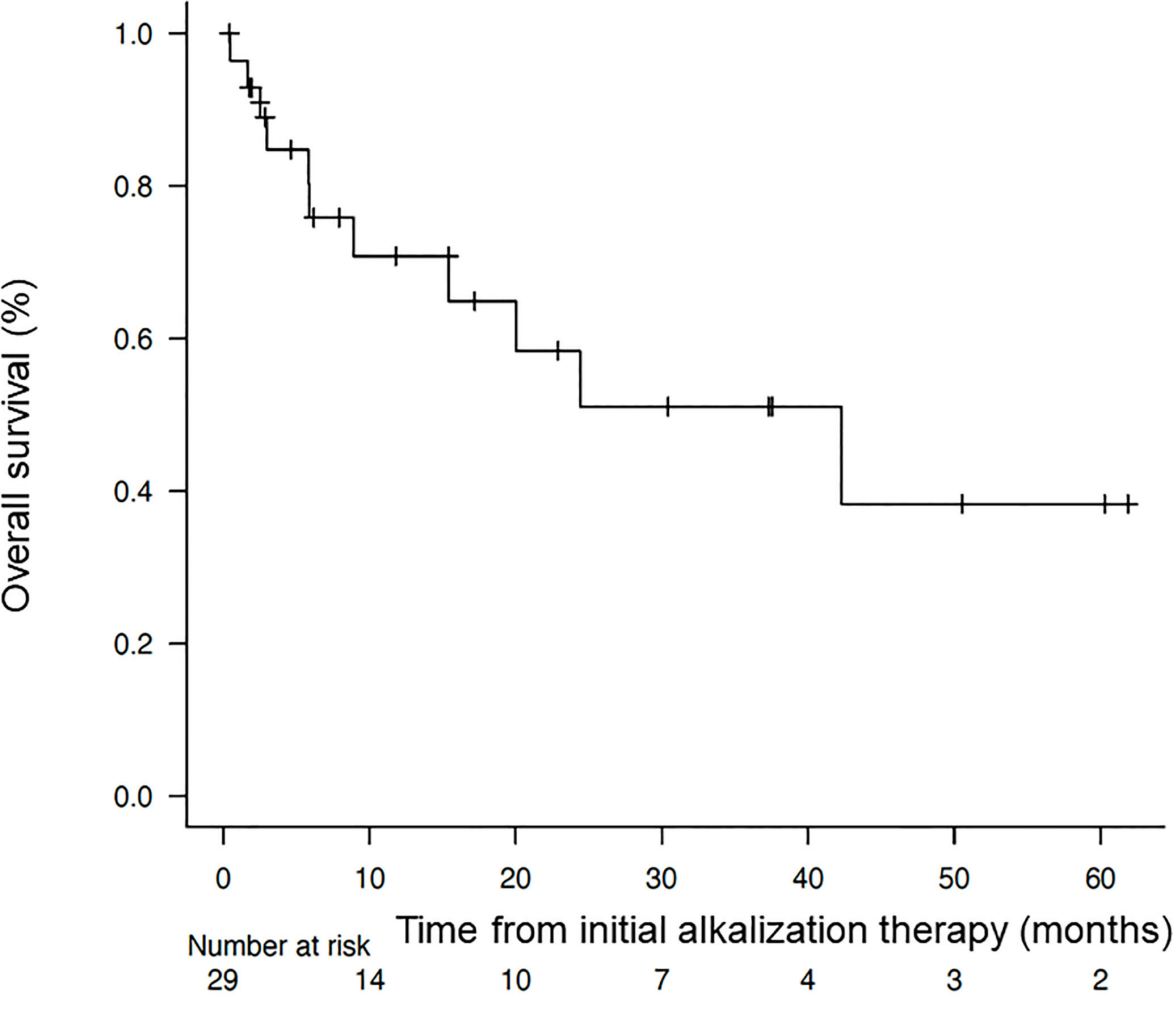
Overall survival of the patients from the start of alkalization therapy. Kaplan–Meier curve of the overall survival of the patients from the start of alkalization therapy.

The median OS from the time of diagnosis was 95.6 months (95% CI = 24.7-not reached), and the median OS from the start of alkalization therapy was 42.3 months (95% CI = 8.93-not reached), as shown in **Figures 2**, **3**.

**Figure 4:**
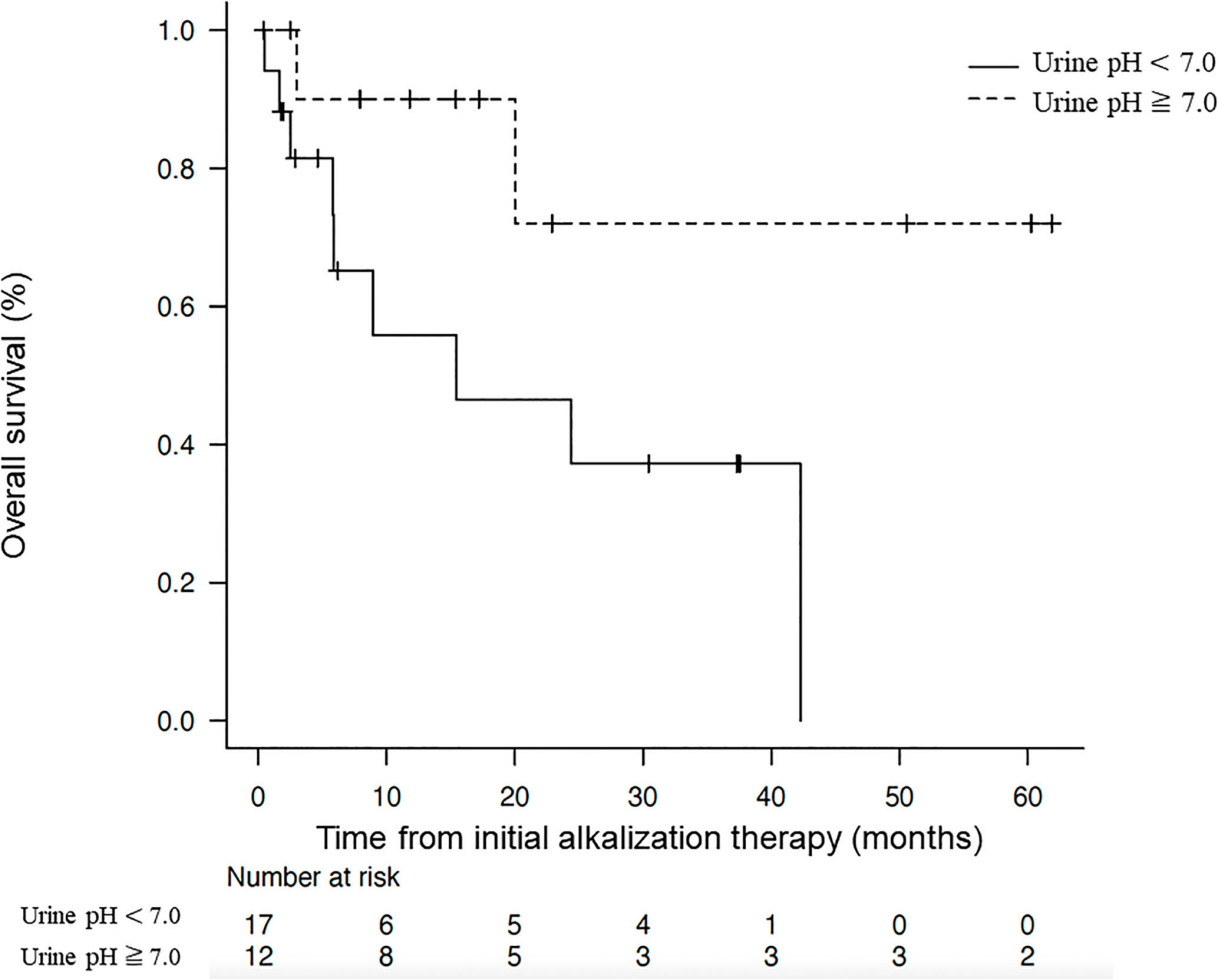
Association between overall survival and urine pH. Kaplan–Meier curves of the overall survival from the start of alkalization therapy between patients with a mean urine pH of ≥ 7.0 and those with a mean urine pH of < 7.0.

The Kaplan–Meier curves of OS from the start of alkalization therapy of patients with a mean urine pH of ≥ 7.0 and those with a mean urine pH of < 7.0 are shown in **Figure 4**. The median OS from the start of alkalization therapy of patients with a mean urine pH of ≥ 7.0 was not reached (n =12, 95% CI = 3.0-not reached), and was significantly prolonged compared with that of patients with a mean urine pH of < 7.0 (15.4 months, n = 17, 95% CI = 5.8-not reached; *p* < 0.05).

## Discussion

HCC often develops in the presence of underlying irreversible liver disease, and hence there is a high frequency of recurrence, and a radical cure is difficult. Current treatment options include surgery, local control therapies, such as TACE/TAE, RFA, irradiation, systemic chemotherapy, and antiviral therapy to inhibit the growth potential of tumors. However, although significant extension in OS has been achieved with these therapies (17), a significant increase in the survival rate of patients has not been achieved and further improvement in outcomes through the development of new treatment options is desired.

Solid tumors other than HCC that are in their advanced clinical stages are often refractory to surgery, systemic chemotherapy, and irradiation. In our clinic, we have reported clinical cases of patients with cancers other than HCC in whom treatment with alkalization therapy has overcome the limitations of conventional treatment (8, 9, 11). In the present study, a single-center, retrospective, observational study was conducted to determine whether an extension in OS is also observed upon alkalization therapy in patients with HCC.

In theory, there are various factors that promote tumor growth, one of which is the TME (18). The energy metabolism of cancer cells becomes predominantly glycolytic, which is called the Warburg effect (19), and this results in the excretion of protons from tumor cells, leading to an acidic TME (20). The acidic environment of the TME causes inflammatory cell infiltration, leading to a worsening of chronic inflammation (21). This results in a vicious cycle of increased vascularity and blood flow imbalance within the tumor, leading to chronic hypoxia and a further increase in glycolytic metabolism in the tumor (19). Basic science studies have reported that the pH of areas of normal cells surrounding the tumor is about 7.2 to 7.4, whereas the pH of tumor cells is decreased to 6.6 to 7.0 (22–25). The goal of alkalization therapy is to interrupt this vicious cycle by increasing the pH of the TME, thereby achieving an antitumor effect (26–28). Our present clinical study was performed on the basis of a significant paradigm shift to consider not only the tumor cells themselves, but also the TME as an important treatment target for malignant tumors.

Regarding studies on the use of drugs targeting the TME, proton pump inhibitors (PPIs) have been reported as an effective treatment option. Preclinical and clinical studies have confirmed that PPIs prevent tumor cells from acquiring resistance to cytotoxic anticancer drugs, and induce apoptosis in tumors in cell culture and in mouse models of malignant melanoma, adenocarcinoma, lymphoma, multiple myeloma, and osteosarcoma (29–34). Clinical studies have also demonstrated increased anti-tumor effects when PPIs are combined with other treatments for metastatic breast and lower gastrointestinal cancers (35, 36). Even in cell culture models of HCC, PPIs have been demonstrated to exert inhibitory effects on tumor growth (37). Thus, there are strong preclinical and clinical lines of evidence for the efficacy of PPI combination therapy as an effective treatment and as a pioneer of systemic buffering therapy. Additionally, alkaline water has been reported to inhibit tumor growth in mouse models of melanoma and prostate cancer (38, 39), and has been shown to extend survival when used in combination with conventional chemotherapy in dogs and cats with cancer (40). However, various issues have prevented the use of both PPIs and alkaline water in our clinic in Japan. For example, regarding PPIs, their use as oral treatments for tumors is not covered by insurance in Japan, and they are therefore not a realistic treatment. As an alternative, we considered dietary improvements, as well as the use of baking soda and citric acid. Regarding alkaline water, there are two methods of generating it: one involves dissolving an alkalizing agent in water, whereas the other involves passing water through an ion exchange membrane. The former uses the commercially available Basenpulver®, which contains multiple inorganic compounds and is not substantially different from the baking soda and citric acid used in this study. Moreover, the fact that Basenpulver® is a mixture makes it difficult to interpret the clinical results. Additionally, there are no widely recognized products similar to Basenpulver® available in Japan, making it unsuitable for clinical use. Regarding the latter method, installing devices in each patient’s home and conducting research would be very costly, and regulating the amount of alkaline water consumed would be difficult. Therefore, we considered an alkalizing diet, as well as the oral intake of baking soda and citric acid, as alternative low-cost approaches that are easy to implement in Japan. In particular, an alkalizing diet is similar to the traditional vegetarian and fish-based diets that have long been consumed in Japan, and is highly compatible with the general eating habits of the majority of our patients.

All 29 patients in this study were instructed to follow an “alkaline diet,” but only 12 were able to achieve and maintain a mean urine pH of 7 or higher. These results suggest that alkalization therapy can increase urine pH, but the degree of the increase may vary among patients. One possibility is that there is an interaction between the clinical condition of each patient and the status of other therapeutic interventions, and the other is that either the diet instructed is not followed strictly, or the diet is followed but is not able to achieve an increase in urine pH.

The median OS from diagnosis of the 29 patients who underwent alkalization therapy was 95.6 months, which was markedly longer than the median OS of 59.66 months for all HCC patients in Japan from 2002 to 2011 (17). This indicates that the implementation of alkalization therapy may have contributed to this extension in OS, despite the difference in the time period of the studies.

Whereas the median OS from the start of alkalization therapy of all 29 patients was 42.3 months, the median OS of patients with a mean urine pH of < 7.0 was 15.4 months, and that of patients with a mean pH of ≥ 7.0 was not reached. This suggests that maintaining a urine pH of ≥ 7.0 with alkalization therapy may have contributed to the extension in OS. Conversely, the results suggest that unless a urine pH of ≥ 7.0 is achieved by alkalization therapy, the treatment may not be effective.

There are several limitations to this study. First, it is a single-center, retrospective, observational study with a small number of cases. It would be desirable to unify the protocol of “alkalization therapy”, accumulate additional cases at multiple centers, and conduct a prospective cohort study. Second, there is wide variation in the patients’ backgrounds. It is preferable to reach conclusions after adjusting background factors, such as sex, clinical tumor stage, presence or absence of extrahepatic lesions, and other therapeutic interventions. Third, comparison with a nonintervention group was not possible. All patients in this study underwent alkalization therapy, and therefore, there was no comparison with a group of patients who did not receive alkalization therapy. The fourth point is the use of urine pH as a target marker for treatment. The pH values of body fluids, including urine and blood, are affected by numerous factors, and it is assumed that variations in drug treatments of the individual patients affected their urine pH. Additionally, it is reasonable to consider that the effect of the tumor on urine pH may increase with increasing malignancy (quality) of the tumor and increasing number of tumor cells (quantity) in each patient. However, even if there are interfering factors that affect urine pH, we believe that therapeutic effects can be obtained by aiming for an alkalization therapy that cancels or overcomes the interference. In the future, it will also be necessary to evaluate the urine pH of patients before intervention with alkalization therapy, according to their degree of tumor progression and tissue type. Finally, there is the possibility that factors other than alkalization therapy may contribute to the extension in OS. Although the alkalization therapy was generally similar for all patients, the specific details of the treatment were inconsistent because the diet is decided by the patients themselves, even if subtle adjustments of drugs are performed and dietary guidance is provided. However, there is currently no data on the comparative effectiveness of the three alkalization approaches (i.e., alkaline diet, oral ingestion of baking soda, and oral ingestion of citric acid), or their synergistic effects. In the future, it will be beneficial to design studies to investigate the optimal amount of baking soda or citric acid to use, and to evaluate the individual effects of each approach, as such studies would further enhance the development of effective alkalizing agents.

## Conclusions

We demonstrated that the addition of alkalization therapy to standard therapies may be associated with more favorable outcomes in HCC patients with increased urine pH after alkalization therapy. Further studies are required to investigate whether alkalization therapy actually contributes to alkalizing the

## Data availability statement

The raw data supporting the conclusions of this article will be made available by the authors, without undue reservation.

## Ethics statement

The studies involving human participants were reviewed and approved by The Japan-Multinational Trial Organization. The patients/participants provided their written informed consent to participate in this study. Written informed consent was obtained from the individual(s) for the publication of any potentially identifiable images or data included in this article.

## Author contributions

MI performed the literature review and wrote the article. RH wrote and supervised the study. RN, HM, and HW performed the acquisition of data. All authors contributed to the article and approved the submitted version.
