# Corrigendum: Effects of alkalization therapy on hepatocellular carcinoma: a retrospective study

**DOI:** 10.3389/fonc.2023.1291026

**Published:** 2023-09-20

**Authors:** Masahide Isowa, Reo Hamaguchi, Ryoko Narui, Hiromasa Morikawa, Hiromi Wada

**Affiliations:** Japanese Society on Inflammation and Metabolism in Cancer, Nakagyo-ku, Kyoto, Japan

**Keywords:** hepatocellular carcinoma, cancer metabolism, alkalization therapy, tumor microenvironment, urine pH

In the published article

35Wang BY, Zhang J, Wang JL, Sun S, Wang ZH, Wang LP, et al. Intermittent high dose proton pump inhibitor enhances the antitumor effects of chemotherapy in metastatic breast cancer. J Exp Clin Cancer Res. 2015;34(1):85.

36Falcone R, Roberto M, D’Antonio C, Romiti A, Milano A, Onesti CE, et al. High-doses of proton pump inhibitors in refractory gastro-intestinal cancer: A case series and the state of art. Dig Liver Dis. 2016;48(12):1503-5.

37Kuchuk O, Tuccitto A, Citterio D, Huber V, Camisaschi C, Milione M, et al. pH regulators to target the tumor immune microenvironment in human hepatocellular carcinoma. Oncoimmunology. 2018;7(7):e1445452.

was not cited in the article. The citation has now been inserted in **Discussion**, P4 and should read:

“Regarding studies on the use of drugs targeting the TME, proton pump inhibitors (PPIs) have been reported as an effective treatment option. Preclinical and clinical studies have confirmed that PPIs prevent tumor cells from acquiring resistance to cytotoxic anticancer drugs, and induce apoptosis in tumors in cell culture and in mouse models of malignant melanoma, adenocarcinoma, multiple myeloma, and osteosarcoma lymphoma(29-34). Clinical studies have also demonstrated increased anti-tumor effects when PPIs are combined with other treatments for metastatic breast and lower gastrointestinal cancers(35, 36). Even in cell culture models of HCC, PPIs have been demonstrated to exert inhibitory effects on tumor growth(37). Thus, there are strong preclinical and clinical lines of evidence for the efficacy of PPI combination therapy as an effective treatment and as a pioneer of systemic buffering therapy. Additionally, alkaline water has been reported to inhibit tumor growth in mouse models of melanoma and prostate cancer(38, 39), and has been shown to extend survival when used in combination with conventional chemotherapy in dogs and cats with cancer(40). However, various issues have prevented the use of both PPIs and alkaline water in our clinic in Japan. For example, regarding PPIs, their use as oral treatments for tumors is not covered by insurance in Japan, and they are therefore not a realistic treatment. As an alternative, we considered dietary improvements, as well as the use of baking soda and citric acid. Regarding alkaline water, there are two methods of generating it: one involves dissolving an alkalizing agent in water, whereas the other involves passing water through an ion exchange membrane. The former uses the commercially available Basenpulver^®^, which contains multiple inorganic compounds and is not substantially different from the baking soda and citric acid used in this study. Moreover, the fact that Basenpulver^®^ is a mixture makes it difficult to interpret the clinical results. Additionally, there are no widely recognized products similar to Basenpulver^®^ available in Japan, making it unsuitable for clinical use. Regarding the latter method, installing devices in each patient’s home and conducting research would be very costly, and regulating the amount of alkaline water consumed would be difficult. Therefore, we considered an alkalizing diet, as well as the oral intake of baking soda and citric acid, as alternative low-cost approaches that are easy to implement in Japan. In particular, an alkalizing diet is similar to the traditional vegetarian and fish-based diets that have long been consumed in Japan, and is highly compatible with the general eating habits of the majority of our patients.”

The authors apologize for this error and state that this does not change the scientific conclusions of the article in any way. The original article has been updated.

In the published article, there was an error. Several words were omitted.

A correction has been made to **Discussion**, P4. This sentence previously stated:

“Preclinical and clinical studies have confirmed that PPIs prevent tumor cells from acquiring resistance to cytotoxic anticancer drugs, and induce apoptosis in tumors in cell culture and in mouse models of malignant melanoma, adenocarcinoma, and lymphoma (29-34).”

The corrected sentence appears below:

“Preclinical and clinical studies have confirmed that PPIs prevent tumor cells from acquiring resistance to cytotoxic anticancer drugs, and induce apoptosis in tumors in cell culture and in mouse models of malignant melanoma, adenocarcinoma, lymphoma, multiple myeloma, and osteosarcoma (29-34).”

The authors apologize for this error and state that this does not change the scientific conclusions of the article in any way. The original article has been updated.

